# Annotations

**Published:** 1891-08-15

**Authors:** 


### Annotations.

**Our Children's Ears.** It is amusing to note to how great an extent the discussion concerning large and protruding ears is occupying the public mind at the present time. Theories as to the origin of the evil are numerous and various. One correspondent to the *Daily Telegraph* boldly makes evolution responsible. He said: "The evolution of an organ is through its use. Why is the giraffe so tall, but because he was for ever trying after the top leaf of the tree; so our young people are getting big ears because they are such busy listeners. Gossip, curiosity, the anxiety to know, the earnest search after news, that accounts for the long ears of the present generation." For a considerable time past I have been much struck by the defect in question amongst rural populations (sad to relate, my observations have been largely made in church), and this fact does not tend to strengthen the argument of our correspondent. It is not amongst this class of the community that we usually look for that keen curiosity and intelligence which he quotes, and we hope he is mistaken in his sign of civilization. The theory of laxity of attention to the matter in youth is much more plausible. Our ancestors certainly paid more attention to the details of personal appearance than we do to-day. When the little nightcaps worn by children of bygone generations went the way of all fashions, we hear of careful parents who doctored ears that promised to protrude beyond the limit of the becoming, by a gentle but judicious "strapping down" at night. Thus the small and shapely ear was more often seen in former times than now—and in the gentle born especially, more care naturally being bestowed upon their ancestors and upon them, than time would permit amongst the busier classes. But at all times natural proclivities will assert themselves. Large feet and hands are not confined to members of the working population, and should the theory of evolution be a sound one, its progress is not to be traced by hundreds, but by tens of hundred years. As the effect appears to be from physical rather than mental causes, should some such simple method as of the ear-cap at night be adopted for children, the future generation will probably benefit by being the possessors of more ornamental, if not of more useful organs of hearing.

**Holidays.** One of the comic papers has an amusing sketch showing John Bull surrounded by his family and trunks starting for the seaside. He is represented as saying: "I don't want to go. I have no money to pay for going, but they insist on it." This, no doubt, represents the average condition of mind of the head of many households in this country this year. It is not uninteresting to watch the debate in the family circle as to where and when. The locality is always more or less of a difficulty, seeing that the father usually holds that one seaside place is exactly like another, and that if the accommodation at Muddleton-in-the-Hole was good last year, the best thing to do is to go there again. The mother and daughters, on the other hand, wish to seek fresh fields and pastures new: nothing short of a new seaside place every year will satisfy them. We believe it would be an economy to the heads of households if they were to charter a caravan and take their families to every watering-place in the country. This could readily be done by a little arrangement in three years, and then the male bird would have peace. Equal difficulties arise when the time of departure is to be determined. The ladies think that it is not fashionable to go until a certain date; the father probably argues that the weather is sure to be better in July than in other months, and that, therefore, fashion is a minor consideration which can be treated with the contempt it deserves. Formerly the month of August was regarded as the very best for a holiday in these islands, but having regard to the vagaries of the weather this season, despite St. Swithin's, we fancy most people will determine to avoid this month in future. Whether the weather has anything to do with it or not we do not know, but we have it on reliable authority that the fashionable months at the seaside are now May and June. We have called attention to this side of the holidays question as it is a serious one, because to the bread-winner a holiday under the present conditions is very often no holiday at all. For a man to be compelled to go to the seaside with all his family, and to sit there for so many weeks bored to death with little to do and plenty of time to do it in, is not only a cruelty but a mistake. It is a mistake in the sense that it lessens the power of the bread-winner to provide the needful for the family's comfort, and so renders the winter months at home anything but peaceful. It is a cruelty, because a man who really works hard during ten months in the year is entitled, and should secure for himself a thorough rest and change; or, in other words, a good holiday when he gives up his business and seeks relief either in the country or abroad.

**Artizans' Cookery.** The working man, his daily wants and amusements, have become one of the most absorbing topics of to-day. Society seems determined not only to give him a good time all round, but to regulate the quality of his mental and even bodily food. The cookery in the poorer homes of England is no new subject for abuse. Abuse has done this much that it has stimulated the public to endeavour to help those who will not help themselves, and finally technical education has stept in with the enthusiasm common to new movements where there is good soil to work upon. Already School Board primers are appearing, in which the culinary art is shaped into as definite a system as is the course of the three "R's." Doubtless, before very long when we pass the village school the chorus from within will send forth instead of the old-timed "six times six is thirty-six," "Don't put the pudding in until the water boils," "When you boil potatoes, be sure you add the salt," or some such golden maxims from the law book of culinary science. But the same fault may appear in these new paths to knowledge, which have distinguished our English methods of education in the past. We may hamper application by a too elaborate theoretical system, and so render all the teaching practically useless. Unless the various conditions of each locality be considered as to facilities of procuring certain kinds of food rather than others, and unless the utensils used in tuition be the same as will be within easy reach of the scholars when they come to put the lessons into practice in their own homes, the result of all the teaching will be a worse failure than before. Instruction of this kind cannot be too definite, for experience shows that even from those practising cooking as a profession, a very small amount of intelligence as apart from knowledge can be depended upon. Neither should it be overlooked that the culinary art is unfortunately placed by custom in the hands of that class of the community which is most indifferent to the subject in its practical application. Cookery is in fact a study, as little congenial to many women as the stiffer paths of knowledge are said to be to the majority.

**Hospitals and Telephones.** Mr. Davy, of the Westminster Hospital, has called attention to the injury inflicted upon patients who are sometimes brought to a large London Hospital where they are refused admission for want of room. A system of hospital inter-communication by telephone similar to that which prevails in Paris, New York, and other cities would spare a patient the danger, anxiety, and uncertainty of seeking admission to a hospital which is already full. Mr. Davy records the following instance as showing the necessity for such inter-communication in London at the present time. A boy broke his leg in St. James's Park, and was carried by a workman to the Westminster Hospital. Many of the surgical wards were closed for necessary repairs, and all the available beds were occupied, so that the boy could not be admitted. With a view to preventing the boy's simple fracture becoming a compound one, the house surgeon applied an outside and inside splint, and recommended his being taken to St. Thomas's Hospital. As the boy's parents resided near the Strand, he was taken in an omnibus to Charing Cross Hospital. There he was refused admission because he had been first attended to at Westminster. From Charing Cross the boy was taken in a cab to King's College Hospital, and was again refused admission for want of room. The parents then took the boy home, and on the next day they again took him to Westminster, where the authorities decided to dismiss a patient who was fairly convalescent, in order to make a bed for this case. We hope that the secretaries of the various London hospitals will take steps to call a conference, and by placing themselves in communication with the National Telephone Company, speedily arrange for a system of telephone inter-communication between all the principal hospitals, and so obviate much avoidable sufferings to many poor people.

